# Rapamycin downregulates α-klotho in the kidneys of female rats with normal and reduced renal function

**DOI:** 10.1371/journal.pone.0294791

**Published:** 2023-11-28

**Authors:** Azahara Espartero, Angela Vidal, Ignacio Lopez, Ana I. Raya, Mariano Rodriguez, Escolastico Aguilera-Tejero, Carmen Pineda

**Affiliations:** 1 Department of Animal Medicine and Surgery, University of Cordoba, Campus Universitario Rabanales, Cordoba, Spain; 2 Maimonides Biomedical Research Institute of Cordoba (IMIBIC), Reina Sofia University Hospital, University of Cordoba, Cordoba, Spain; Max Delbruck Centrum fur Molekulare Medizin Berlin Buch, GERMANY

## Abstract

Both mTOR and α-klotho play a role in the pathophysiology of renal disease, influence mineral metabolism and participate in the aging process. The influence of mTOR inhibition by rapamycin on renal α-klotho expression is unknown. Rats with normal (controls) and reduced (Nx) renal function were treated with rapamycin, 1.3 mg/kg/day, for 22 days. The experiments were conducted with rats fed 0.6% P diet (NP) and 0.2% P diet (LP). Treatment with rapamycin promoted phosphaturia in control and Nx rats fed NP and LP. A decrease in FGF23 was identified in controls after treatment with rapamycin. In rats fed NP, rapamycin decreased mRNA α-klotho/GADPH ratio both in controls, 0.6±0.1 vs 1.1±0.1, p = 0.001, and Nx, 0.3±0.1 vs 0.7±0.1, p = 0.01. At the protein level, a significant reduction in α-klotho was evidenced after treatment with rapamycin both by Western Blot: 0.6±0.1 vs 1.0±0.1, p = 0.01, in controls, 0.7±0.1 vs 1.1±0.1, p = 0.02, in Nx; and by immunohistochemistry staining. Renal α-klotho was inversely correlated with urinary P excretion (r = -0.525, p = 0.0002). The decrease in α-klotho after treatment with rapamycin was also observed in rats fed LP. In conclusion, rapamycin increases phosphaturia and down-regulates α-klotho expression in rats with normal and decreased renal function. These effects can be observed in animals ingesting normal and low P diet.

## Introduction

Rapamycin, a macrolide antibiotic produced by *Streptomyces hygroscopicus*, is the classic inhibitor of the mTOR (molecular target of rapamycin) pathway that integrates nutrient signals to control energy metabolism [1]. mTOR is found in two distinct protein complexes: mTORC1, which is inhibited by rapamycin, and mTORC2, that is thought not to be affected by rapamycin (although recent data suggest that it may also be sensitive after a prolonged exposure) [2, 3]. Rapamycin has immunosuppressive properties based on its ability to interfere with lymphocyte growth and differentiation [4] and it is currently used to prevent rejection after kidney transplantation [5].

Activation of the mTOR pathway is thought to participate in the pathophysiology of renal disease [6, 7], particularly in diabetic kidney disease [8]. Lloberas et al. demonstrated that mTOR blockade by rapamycin slowed the progression of diabetic kidney disease in rats by reducing albuminuria and improving renal histopathology [9]. In another study, rapamycin reduced glomerulonephritis and IgG deposition in the subendothelial tuft of diabetic mice [10]. However, rapamycin has also been shown to aggravate podocyte injury in experimental nephropathies [11] and in transplant patients [12].

Rapamycin also has effects on mineral metabolism -e.g. rapamycin is known to promote phosphaturia in healthy mice by inhibiting renal tubular transport of phosphate (P) [13]. This is so despite the fact that recent work has demonstrated that treatment with rapamycin decreases the production of the phosphaturic hormone fibroblast growth factor 23 (FGF23) by cultured bone cells [14].

In addition, downregulation of the mTOR pathway may be involved in slowing aging and increasing lifespan [15]. In fact, mTOR inhibition is believed to play a major role in the protective effects of caloric restriction on aging and rapamycin is being advocated as an anti-aging drug [16].

α-Klotho, a protein that is produced predominantly in the kidneys, has actions closely linked to those of mTOR, namely: a) it is related to kidney disease—α-klotho levels have been reported to decrease in a variety of renal disorders and α-klotho deficiency is known to facilitate progression of kidney disease [17]; b) it plays a major role in the regulation of phosphaturia—α-klotho is an essential cofactor for the phosphaturic effect elicited after the interaction of FGF23 with its receptor FGFR1 [18] and functions as an autocrine enzyme that directly promotes phosphaturia [19]; and, c) it participates in the aging process—decreased α-klotho expression results in accelerated aging and α-klotho overexpression extends lifespan in mice [20]. Thus, it seems reasonable that α-klotho may be connected to mTOR and consequently may be regulated by rapamycin.

There is very little information on the effect of mTOR inhibition by rapamycin on renal α-klotho expression and the data available, which is to some extent contradictory, has been obtained mainly in renal transplant recipients who are receiving a variety of drugs [21–24]. Thus, the objective of this study was to investigate the influence of rapamycin treatment on renal α-klotho expression and related parameters affecting P metabolism in rats with normal and reduced renal function. A secondary objective, raised in the course of the study was to evaluate the effect of rapamycin in rats fed P restricted diets.

## Materials and methods

### Ethics

All experimental protocols were reviewed and approved by the Ethics Committee for Animal Research of the University of Cordoba and by Junta de Andalucia (Spain) (Ethical Code Number 11/11/2021/176, date 15/11/2021). All protocols were carried out in accordance with the approved guidelines. They followed the guiding principle laid down by the Higher Council of Scientific Research of Spain following the normal procedures directing animal welfare and adhered to the recommendations included in the Guide for Care and Use of Laboratory Animals (US Department of Health and Human Services, NIH) and European laws (Art. 41.1, Real Decreto 53/2013, Directive 2010/63/EU of the European Parliament). All animal welfare considerations were taken, including effort to minimize suffering and distress, use of analgesics and anesthetics and special housing conditions. All research personnel was trained and certified for animal experiments.

### Animals and diets

Female Wistar rats provided by the Animal Housing Facilities of the University of Cordoba (Cordoba, Spain) were used in the experiments. Rats, aged 2 months at the beginning of the studies, were housed with a 12h/12h light/dark cycle and were fed a standard diet (Altromin C1031, AltrominSpezialfutter GmbH, Germany) containing normal amounts of calcium (Ca), and vitamin D: 0.6% of Ca and 500 IU/g of vitamin D. The Na content of the diet was 2480 mg/kg and the Cl content 1984 mg/kg. The diet had either normal (0.6%) or low (0.2%) P concentration. Daily food intake was recorded for each rat by averaging the amount of food eaten every week. Health and behaviour were monitored daily. Humane endpoints were used in the study. The specific criteria to determine when animals should be euthanized before ending the study included: complete anorexia, inability or extreme reluctance to stand, signs of severe organ system dysfunction, and pain and/or distress not responsive to analgesics. Animals were scheduled to be sacrificed 24 h after exhibiting any of these signs.

### Induction of kidney disease

Renal function was reduced by nephrectomy with ablation of 5/6 of renal mass (5/6Nx). Before performing surgery, rats were anesthetized using inhaled isoflurane (Isovet, Braun, Barcelona, Spain). 5/6 Nx was performed in two steps. In the first step a 5- to 8-mm incision was made on the left mediolateral surface of the abdomen. The left kidney was exposed, and the two poles (2/3 of renal mass) were ablated. The kidney was inspected and returned to an anatomically neutral position within the peritoneal cavity. The abdominal wall and skin incisions were closed with sutures, and the rat was placed back into its home cage. After 1 week of recovery, in the second step, the animal was reanesthetized, a 5- to 8-mm incision was made on the right mediolateral surface of the abdomen. The right kidney was exposed and unencapsulated, the renal pedicle was clamped and ligated, and the kidney was removed. The ligated pedicle was returned to a neutral anatomical position and the abdomen and skin incisions were closed with suture materials. Fentanyl, 0.2 mg/kg, ip (Fentanest, Kern Pharma, Barcelona, Spain) was used as analgesic agent.

### Experimental design

Two sets of experiments were conducted. In the first one rats were fed a normal P diet (NP) while in the second experiment rats were fed a diet with low P content (LP). The design was identical in both experiments. Thus, in each experiment, rats with intact renal function and with reduced renal function (Nx) were allotted to 2 groups (n = 16–20). Half the rats received placebo treatment (NP, NxNP, LP and NxLP) and the other half were treated with rapamycin (Rap) at a dose of 1.3 mg/kg/day (NP-Rap, NxNP-Rap, LP-Rap and NxLP-Rap) (Supplementary data, S1 Fig).

Rapamycin (Rapamune, Pfizer Europe, Belgium) was administered daily in unflavored gelatin tablets (80 mg of pure gelatin dissolved in 1 ml of distilled water). The placebo groups were fed gelatin tablets without rapamycin. Visual confirmation of complete consumption of the gelatin tablet was always obtained. Rats received the treatments for 22 days. None of the animals reached the humane endpoints and no animal died spontaneously before the end of the experiments. Accordingly, all animals were euthanized at the end of the study. Euthanasia was performed, after overnight fasting, by exsanguination under general anesthesia (inhaled isoflurane). Blood samples, drawn from the abdominal aorta, and kidney samples were collected. Urine samples were obtained in the last 3 days prior to sacrifice by placing the rats in metabolic cages.

### Blood and urine chemistries

Immediately after obtaining the blood sample, plasma was separated by centrifugation and stored at –20°C until assayed. Plasma concentrations of urea, creatinine, Ca, and P as well as urine Ca and P were measured by spectrophotometry (BioSystems SA, Barcelona, Spain). ELISA tests were used to quantify plasma concentrations of intact FGF23 (Kainos Laboratories, Tokyo, Japan), parathyroid hormone (PTH) (Immutopics Inc., Quidel Corporation, OH, USA), and cystatin C (BioVendor—Laboratomí medicína a.s., Brno, Czech Republic).

### RNA extraction and real time Reverse Transcription-Polymerase Chain Reaction (RT-PCR)

Total RNA was isolated from renal tissue using TRIzol reagent protocol (Invitrogen, Waltham, Massachusetts, USA) and a treatment with DNase I amplification Grade (Sigma-Aldrich, St. Louis, MO, USA) was done according to the manufacturer’s instruction. Quantification was performed by spectrophotometry (ND-1000, Nanodrop Technologies, Wilmington, DE, USA). The sequence of primers used for RT-PCR is shown in Supplementary data, S1 Table. Quantification was done using the QuantiTect SYBR Green RT-PCR kit (Qiagen GmbH, Hilden, Germany) for 50 ng of RNA and 1 μl of primer. The mRNA expression was analyzed in the Light Cycler thermal cycler system (Roche Diagnostics, Indianapolis, IN, USA) and the relative expression of the target genes was determined using the 2^-ΔΔ^Ct method.

### Protein extraction and Western blot analysis

Proteins were isolated from renal tissue by using a lysis buffer HEPES (10 mmol/l), KCl (10 mmol/l), EDTA (0.1 mmol/l), EGTA (0.1 mmol/l), DTT (1 mmol/), PMSF (0.5 mmol/l), protease inhibitor cocktail (70 μg/ml), and I-Gepal CA-630 (0.6%), pH 7.9 (Sigma Aldrich, St. Louis, MO, USA). Protein concentration was determined by the Bradford method. For Western blot analysis, 50 μg of protein was electrophoresed on a 4–20% SDS-polyacrylamide gel (Criterion TGX, BioRad, Hercules, CA, USA) and electrophoretically transferred (Transfer Systems, BioRad, Hercules, CA, USA) from the gels onto nitrocellulose membranes (Trans-blot Turbo transfer pack, BioRad, Hercules, CA, USA). The following steps were performed with gentle shaking. Membranes were incubated in Tris-buffered saline with Tween 20 (TBST) solution (20 mM Tris-HCl (pH 7.6), 0.2% Tween 20, 150 mM NaCl) (Sigma Aldrich, St. Louis, MO, USA), and 5% nonfat dry milk (Bio-Rad, Hercules, CA, USA) at room temperature for 1h to avoid nonspecific binding. Membranes were then washed with TBST buffer and incubated overnight at 4°C with an anti-⍺-klotho antibody (KO603; Transgenic, Kobe, Japan). The membranes were then washed with TBST buffer and immunolabeled using a peroxidase-conjugated secondary antibody (1:5000 dilution; Santa Cruz Biotechnology Inc., Santa Cruz, CA, USA). Finally, they were revealed on autoradiographic film using ECL Western Blotting Detection Substrate (Thermo Fisher Scientific, Waltham, MA 02451 USA). GAPDH (sc-59540; Santa Cruz Biotechnology Inc., Santa Cruz, CA, USA) was used as a housekeeping protein to ensure equal loading of the gels. Protein levels were quantified using ImageJ software (National Institutes of Health, Bethesda, MD, USA).

### Immunohistochemistry

Kidney samples were fixed in 10% formalin and embedded in paraffin. Three μm sections were deparaffinized, rehydrated and microwave-treated in 0.01 mmol/l citrate buffer (pH 6) for 20 min. Then, samples were incubated with endogenous enzyme blocking solution (Vector Laboratories, Newark, CA, USA) for 10 min. Sections were blocked with normal horse serum 2.5% (Vector Laboratories, Newark, CA, USA) for 20 min. After that, sections were incubated overnight at 4°C in a humidified chamber with primary mouse anti-⍺-klotho antibody (Alpha Diagnostics, Tilts, Denmark) at 1:100 dilution. After rinsing, the sections were incubated for 30 min at room temperature with the ImmPRESS Universal Antibody polymer reagent (Vector Laboratories, Newark, CA, USA) and treated with diaminobenzidine substrate for 1 min (Vector Laboratories, Newark, CA, USA). Every step was followed by three washes with phosphate-buffered saline for 5 min. Finally, sections were counterstained with haematoxylin (Dako, Santa Clara, CA, USA). Assessment of α-klotho was performed in 8 randomly selected fields per kidney by measuring optic density (units) in renal tubules expressing α-klotho, using ImageJ software (National Institutes of Health, Bethesda, MD, USA).

### Statistics

Values are expressed as the mean ± standard error (SE). The difference between means for two different groups was determined by t-tests; the difference between means for three or more groups was assessed by ANOVA. Fisher LSD test was used as a post-hoc procedure. A correlation study was carried out using the Pearson test. A p<0.05 was considered significant.

## Results

### Experiments with Normal P diet

**Fig 1:**
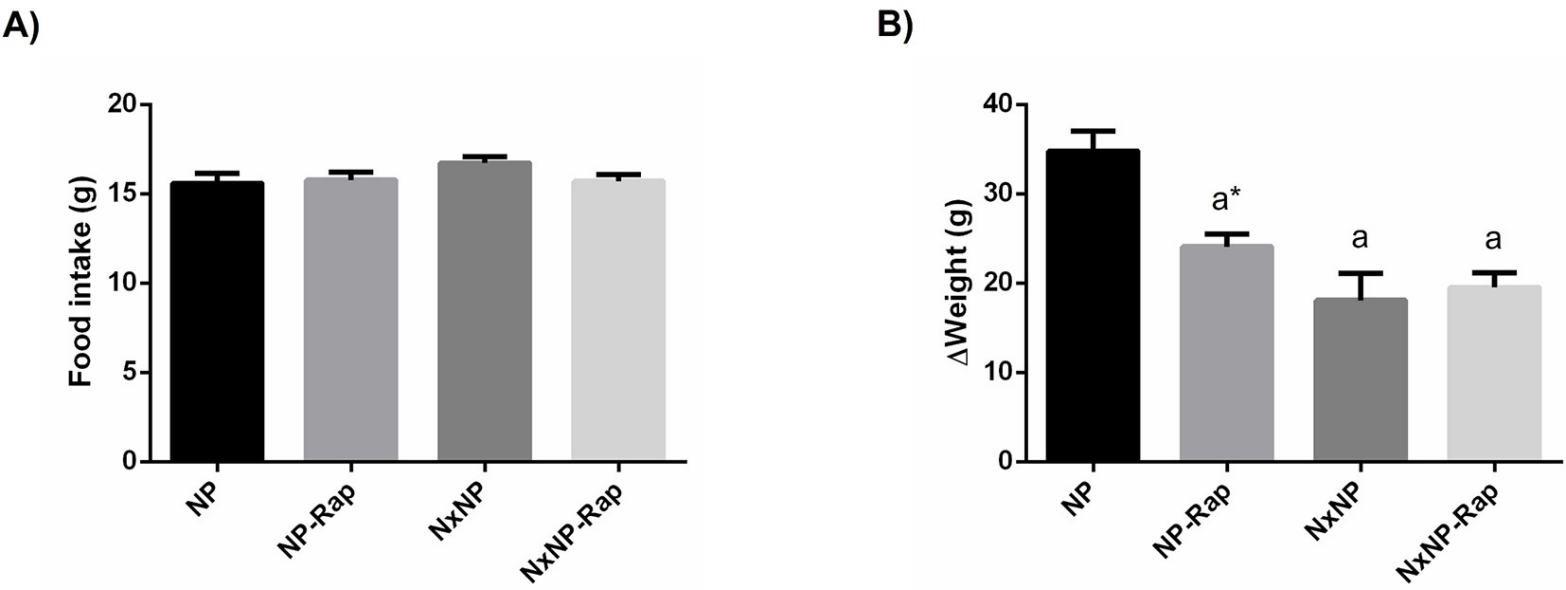
Food intake and changes in body weight in rats fed normal phosphorus. (A) Food intake and (B) changes in body weight in rats fed 0.6% phosphorus with intact (NP) and reduced (NxNP) renal function receiving either placebo or rapamycin (Rap). ^a^*P*<0.05 vs NP; *P<0.05 vs its placebo counterpart.

Although there were no differences in food intake between groups (Fig 1A), in the course of the experiments NxNP rats gained less weight, 18.1 ± 3.0 g, than NP rats, 34.8 ± 2.2 g, p<0.0001. Treatment with rapamycin also reduced body weight gain in rats with intact renal function to 24.1 ± 1.4 g, p<0.001 vs NP (Fig 1B).

**Fig 2:**
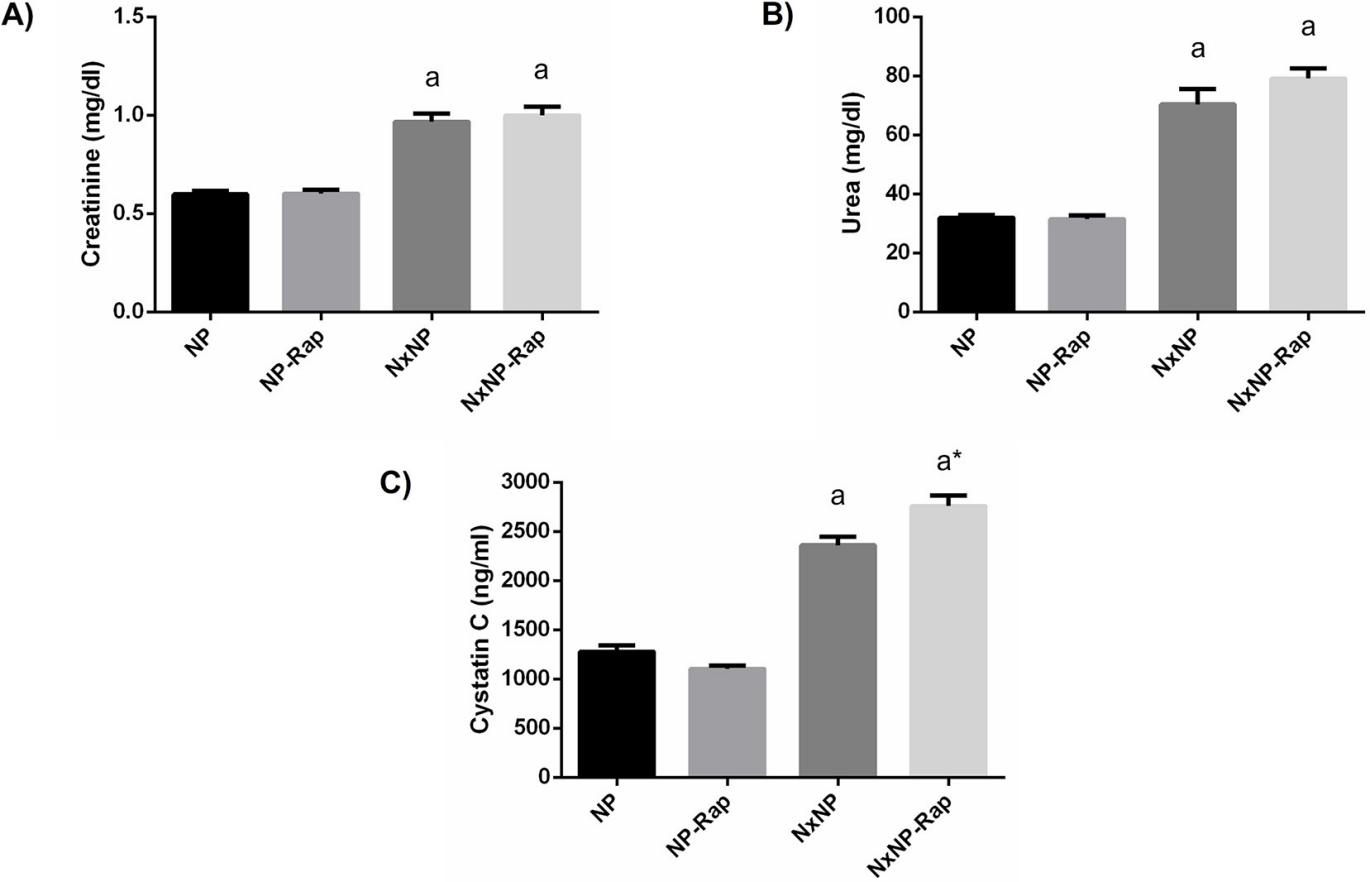
Plasma metabolites related to renal function in rats fed normal phosphorus. (A) Plasma creatinine, (B) urea and, (C) cystatin C concentrations in rats fed 0.6% phosphorus with intact (NP) and reduced (NxNP) renal function receiving either placebo or rapamycin (Rap). ^a^*P*<0.05 vs NP; *P<0.05 vs its placebo counterpart.

An increase in plasma creatinine, 0.97 ± 0.04 mg/dl, urea, 70.4 ± 5.3 mg/dl, and cystatin C, 2362 ± 84 ng/ml, was observed in NxNP rats when compared with NP rats, 0.60 ± 0.02 mg/dl, 31.9 ± 0.9 mg/dl and 1279 ± 63 ng/ml. Rapamycin treatment did not influence renal function parameters in rats with normal renal function and only produced a small increase in cystatin C in Nx rats (Fig 2).

**Fig 3:**
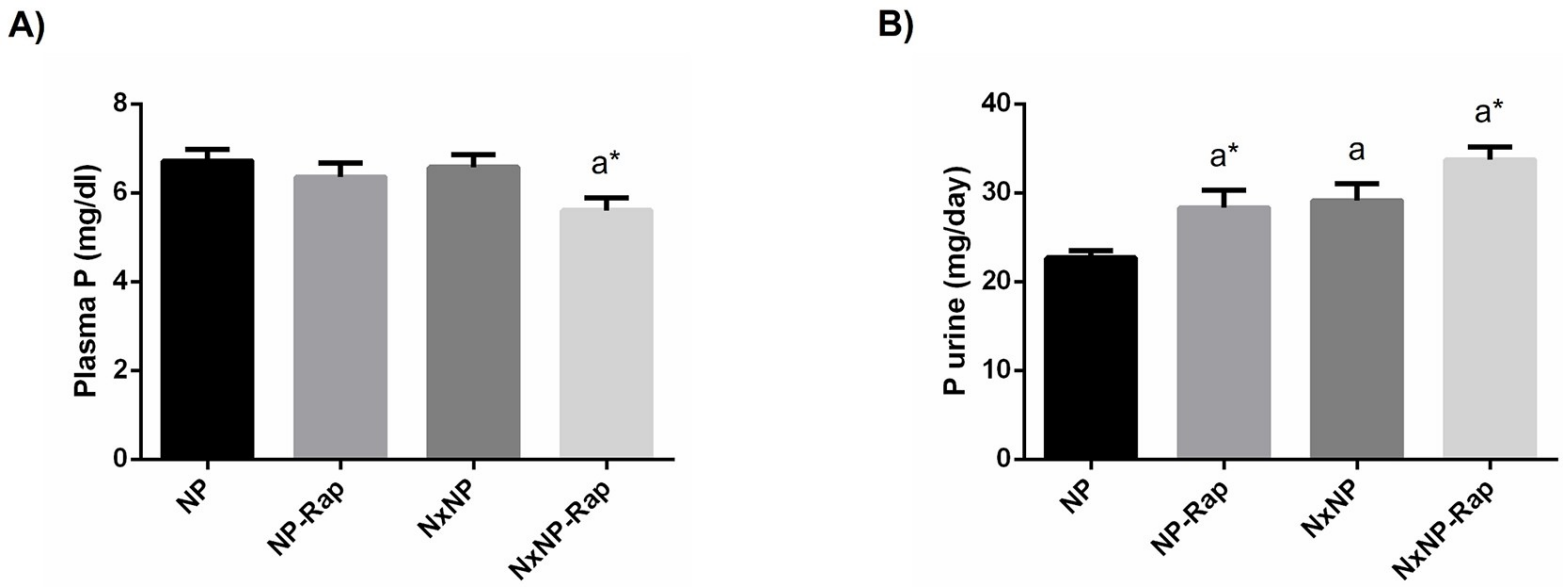
Plasma concentration and urinary excretion of phosphorus in rats fed normal phosphorus. (A) Plasma concentration and (B) daily urinary excretion of phosphorus (P) in rats fed 0.6% phosphorus with intact (NP) and reduced (NxNP) renal function receiving either placebo or rapamycin (Rap). ^a^*P*<0.05 vs NP; *P<0.05 vs its placebo counterpart.

Plasma P concentration was not affected by Nx but tended to be decreased in rats treated with rapamycin and the differences reached significance in Nx rats, 5.6 ± 0.3 vs 6.6 ± 0.3 mg/dl, p = 0.02 (Fig 3A). Urinary excretion of P was higher in NxNP rats, 29.1 ± 1.9 mg/day, as compared with NP rats, 22.7 ± 0.8 mg/day, p = 0.006. Treatment with rapamycin significantly increased phosphaturia both in rats with normal renal function, 28.3 ± 2.0 mg/day, p = 0.01, and in rats with reduced renal function, 33.7 ± 1.4 mg/day, p = 0.04 (Fig 3B).

**Fig 4:**
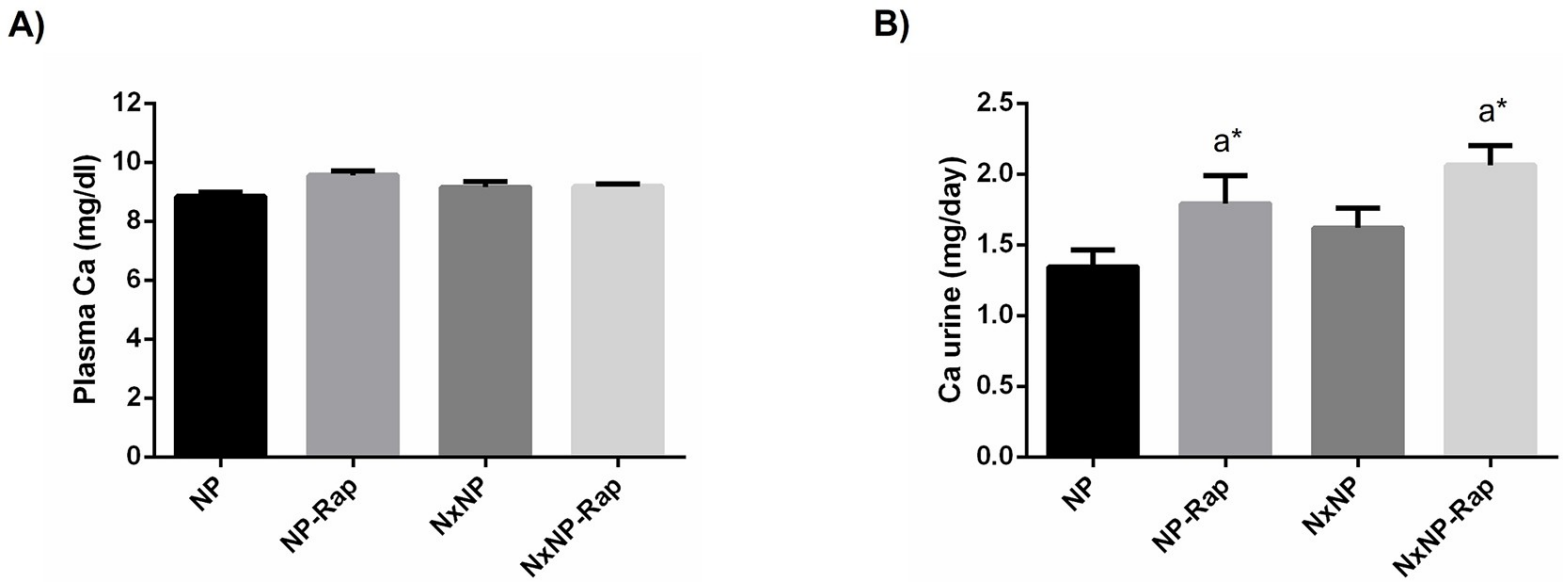
Plasma concentration and urinary excretion of calcium in rats fed normal phosphorus. (A) Plasma concentration and (B) daily urinary excretion of calcium (Ca) in rats fed 0.6% phosphorus with intact (NP) and reduced (NxNP) renal function receiving either placebo or rapamycin (Rap). ^a^*P*<0.05 vs NP; *P<0.05 vs its placebo counterpart.

Plasma Ca concentration did not show intergroup differences (Fig 4A). Changes in urinary excretion of Ca were similar to those observed in P. Nx rats tended to increase urinary Ca excretion but the differences did not reach statistical significance. Treatment with rapamycin increased urinary Ca excretion in rats with normal renal function, 1.8 ± 0.2 mg/day vs 1.3 ± 0.1 mg/day in placebo-treated rats, p = 0.04, and in rats with reduced renal function, 2.1 ± 0.1 mg/day vs 1.6 ± 0.1 mg/day in rats receiving placebo, p = 0.04 (Fig 4B).

**Fig 5:**
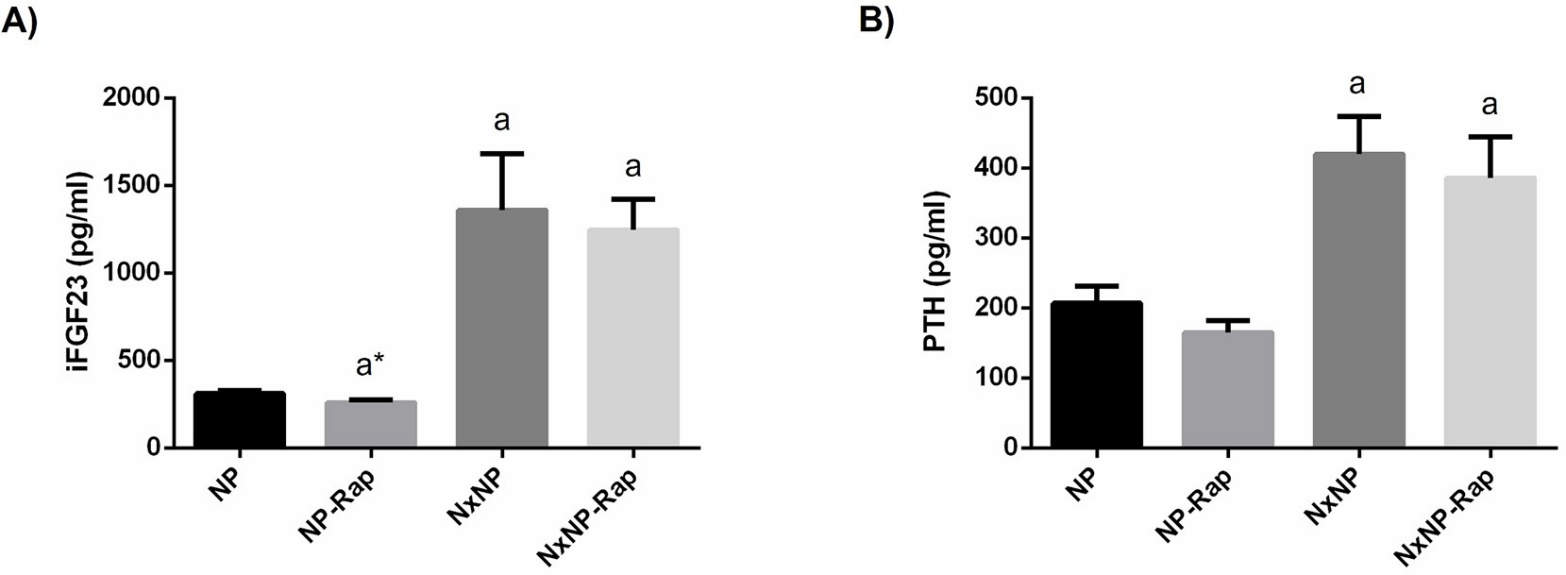
Plasma concentration of intact fibroblast growth factor 23 and parathyroid hormone in rats fed normal phosphorus. (A) Plasma concentration of intact fibroblast growth factor 23 (iFGF23) and (B) parathyroid hormone (PTH) in rats fed 0.6% phosphorus with intact (NP) and reduced (NxNP) renal function receiving either placebo or rapamycin (Rap). ^a^*P*<0.05 vs NP; *P<0.05 vs its placebo counterpart.

Plasma concentrations of FGF23 were much higher in Nx rats, 1358 ± 324 pg/ml, than in rats with intact renal function, 311 ± 17 pg/ml, p = 0.0001. Treatment with rapamycin significantly (p<0.05) reduced FGF23 in rats with intact renal function, 259 ± 16 pg/ml, but not in Nx rats, 1246 ± 176 pg/ml (Fig 5A). An increase in plasma PTH was also found in Nx rats, 419.9 ± 53.8 vs 206.7 ± 24.5 pg/ml, p = 0.0008, but no significant differences were observed in PTH after treatment with rapamycin (Fig 5B).

**Fig 6:**
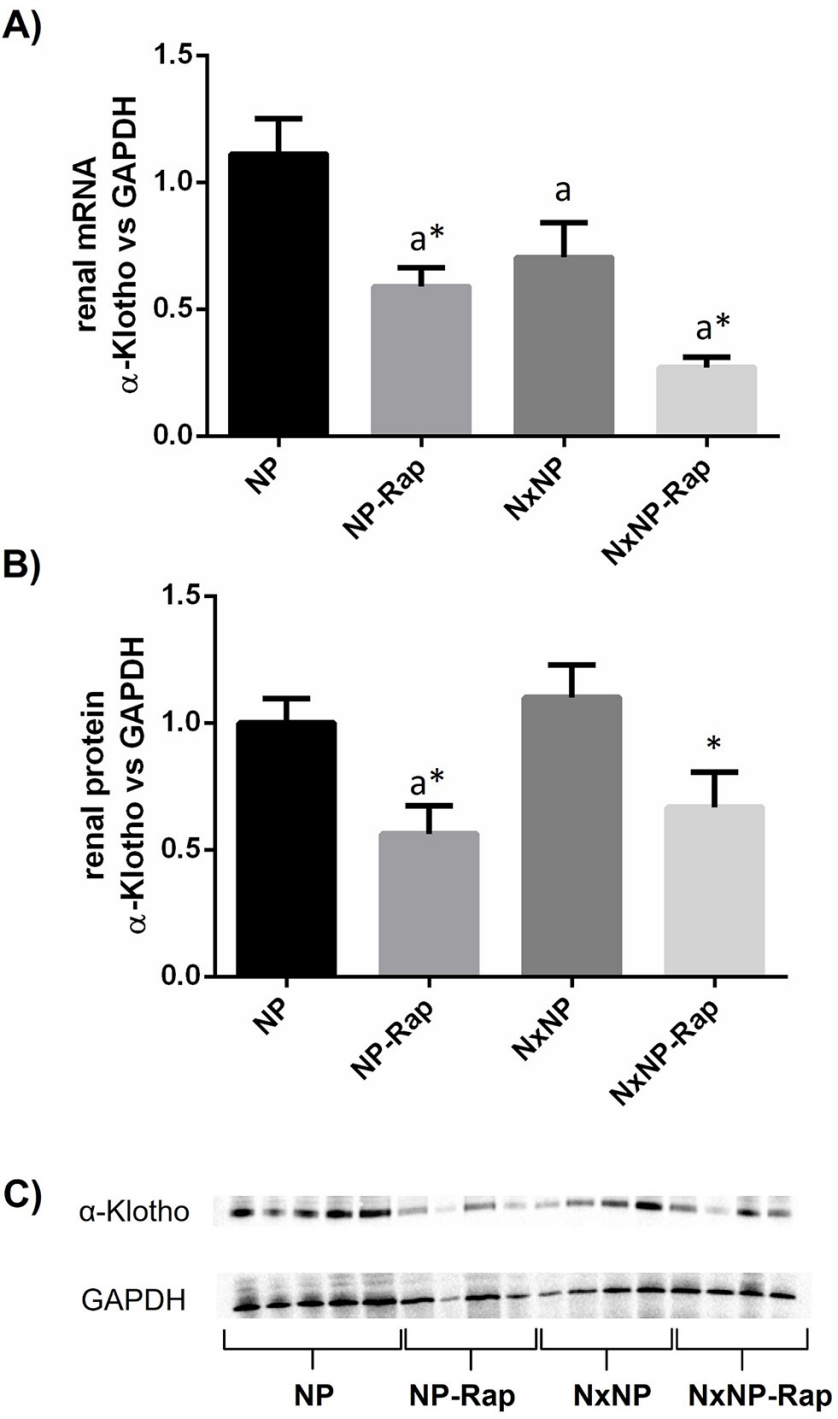
Renal expression of α-klotho at mRNA and protein level and correlation between renal mRNA α-klotho and urine phosphorus in rats fed normal phosphorus. Renal expression of α-klotho at (A) mRNA and (B) protein level in rats fed 0.6% phosphorus with intact (NP) and reduced (NxNP) renal function receiving either placebo or rapamycin (Rap). ^a^*P*<0.05 vs NP; *P<0.05 vs its placebo counterpart. (C) Representative images of Western Blot (the complete Western Blot results are shown as Supplementary Data, S1 Raw images).

**Fig 7:**
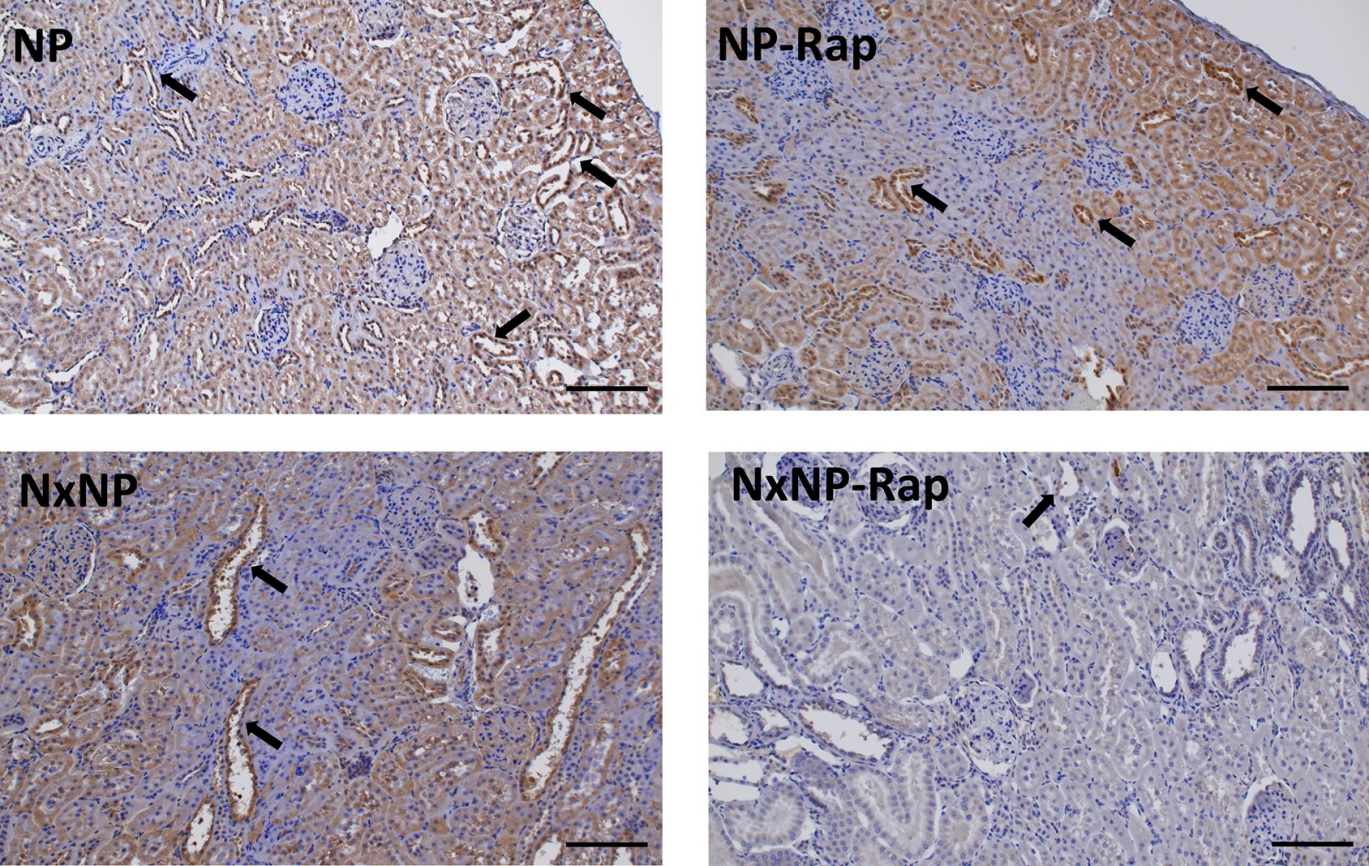
α-Klotho Renal immunohistochemistry. Immunohistochemistry for ⍺-klotho, in renal tissue from rats fed 0.6% phosphorus with intact (NP) and reduced (NxNP) renal function receiving either placebo or rapamycin (Rap). Positive staining (brown color) was located in the cytoplasm of tubular cells of the kidney cortex, with preferential expression in distal tubules (arrows). Scale bar = 100 μm.

Renal α-klotho expression is presented in Fig 6. Nephrectomy resulted in reduced mRNA α-klotho/GADPH ratio, 0.7 ± 01 vs 1.1 ± 0.1, p = 0.01. Treatment with rapamycin decreased mRNA α-klotho/GADPH ratio both in rats with normal renal function, 0.6 ± 0.1, p = 0.001, and in rats with reduced renal function, 0.3 ± 0.1, p = 0.01 (Fig 6A). At the protein level, rapamycin also resulted in a significant reduction in α-klotho in rats with intact renal function, 0.6 ± 0.1 vs 1.0 ± 0.1, p = 0.01, and in Nx rats, 0.7 ± 0.1 vs 1.1 ± 0.1, p = 0.02 (Fig 6B and 6C). The decrease in α-klotho after treatment with rapamycin was corroborated by immunohistochemistry, as shown in Fig 7. α-Klotho staining was less intense in the rapamycin-treated groups, both in rats with intact renal function, 0.08 ± 0.01 vs 0.11 ± 0.001 units, and in nephrectomized rats, 0.06 ± 0.01 vs 0.08 ± 0.01 units. An inverse correlation (r = -0.525, p = 0.0002) was observed between the mRNA α-klotho quantitative data obtained by PCR and the daily urine phosphorus excretion measured by spectrophotometry (Supplementary Data, S2 Fig).

### Experiments with low P diet

To obtain a wider understanding of the role of rapamycin-induced phosphaturia on the observed changes in renal α-klotho expression, additional experiments were performed in rats that had a very low urine P concentration secondary to feeding a diet with low (0.2%) P content.

In these experiments, rapamycin treatment also reduced body weight gain in rats with normal renal function 15.3 ± 23 vs 29.2 ± 3.2 g, p = 0.009, but not in Nx rats 6.2 ± 4.0 vs 10.0 ± 3.3 g.

Changes in biochemical parameters elicited by Nx and by treatment with rapamycin in rats fed LP were in agreement with what had been observed in the experiments with NP. No intergroup differences were observed in either plasma Ca or P. Both FGF23 and PTH were much higher in Nx groups than in rats with normal renal function and rapamycin treatment decreased FGF23 only in rats with intact renal function, 205 ± 19 vs 310 ± 30 pg/ml, p<0.05 (Table 1).

**Table 1:** Plasma biochemical parameters and hormones in the groups fed a low phosphorus diet at the end of the experiment.

	LP	LP-Rap	NxLP	NxLP-Rap
Calcium, mg/dl	9.7 ± 0.1	9.8 ± 0.3	8.2 ± 0.2^a^	9.4 ± 0.2^a^
Phosphorus, mg/dl	7.9 ± 0.7	7.1 ± 0.4	7.7 ± 0.3	6.8 ± 0.3
Creatinine, mg/dl	0.53 ± 0.01	0.59 ± 0.01	0.93 ± 0.04^a^	1.05 ± 0.07^a^
Urea, mg/dl	31.0 ± 1.6	29.7 ± 1.7	62.3 ± 5.1^a^	81.6 ± 7.9^a^*
Cystatin C (ng/ml)	1315 ± 59	1212 ± 27	2496 ± 151^a^	2650 ± 171^a^
FGF23, pg/ml	310 ± 30	205 ± 19^a^*	641 ± 423	1201 ± 319 ^a^
PTH, pg/ml	131.6 ± 15.5	141.3 ± 39.2	386.4 ± 64.0^a^	315.6 ± 73.1

FGF23, fibroblast growth factor 23; LP, rats with intact renal function fed a low phosphorus diet; LP-Rap, rats with intact renal function fed a low phosphorus diet and treated with rapamycin; NxLP, nephrectomized rats fed a low phosphorus diet; NxLP-Rap, nephrectomized rats fed a low phosphorus diet and treated with rapamycin; PTH, parathyroid hormone. Values are means ± SE.

^a^*p* < 0.05 vs LP

*p < 0.05 vs its placebo counterpart.

**Fig 8:**
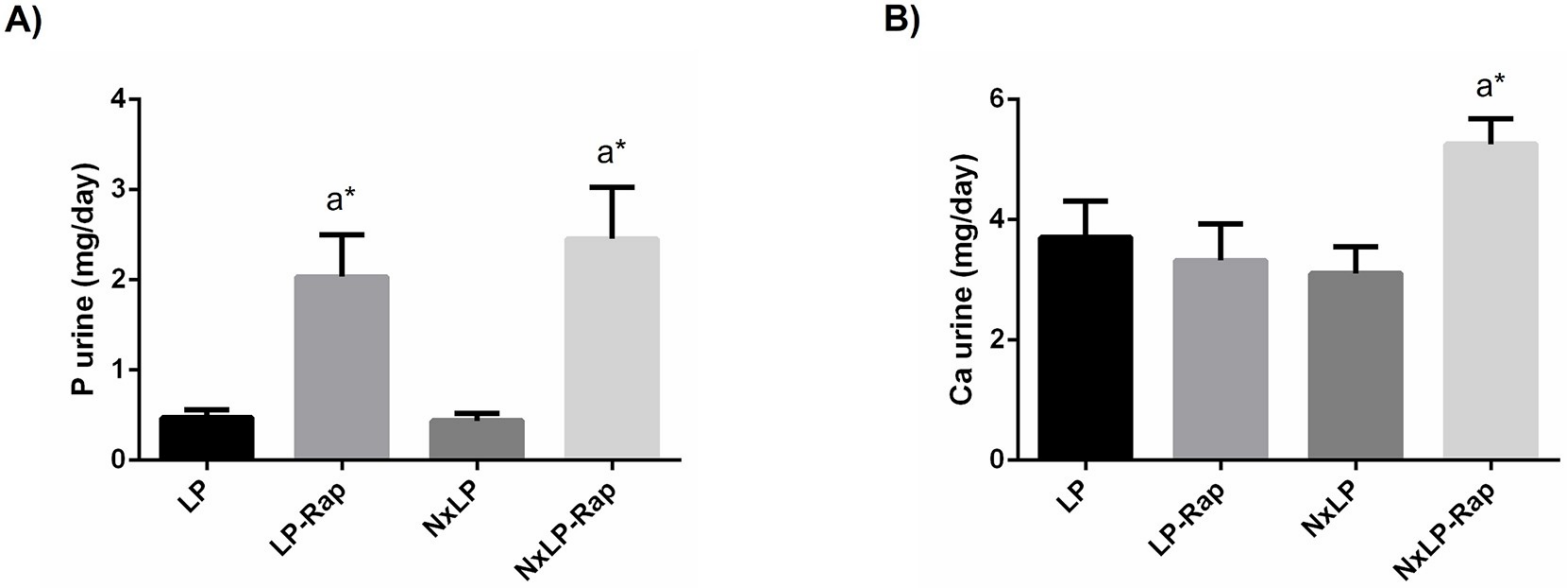
Urinary excretion of phosphorus and calcium in rats fed low phosphorus. (A) Daily urinary excretion of phosphorus (P) and (B) calcium (Ca) in rats fed 0.2% P with intact (LP) and reduced (NxLP) renal function receiving either placebo or rapamycin (Rap). ^a^*P*<0.05 vs NP; *P<0.05 vs its placebo counterpart.

Urinary excretion of P was unaffected by Nx but was significantly increased by rapamycin both in rats with normal renal function, 2.04 ± 0.46 vs 0.47 ± 0.08 mg/day, p = 0.04, and in Nx rats, 2.46 ± 0.57 vs 0.44 ± 0.08 mg/day, p = 0.009. Urinary excretion of Ca was increased only in Nx rats receiving rapamycin, 5.26 ± 0.42 vs 3.11 ± 0.44, p = 0.02 (Fig 8).

**Fig 9:**
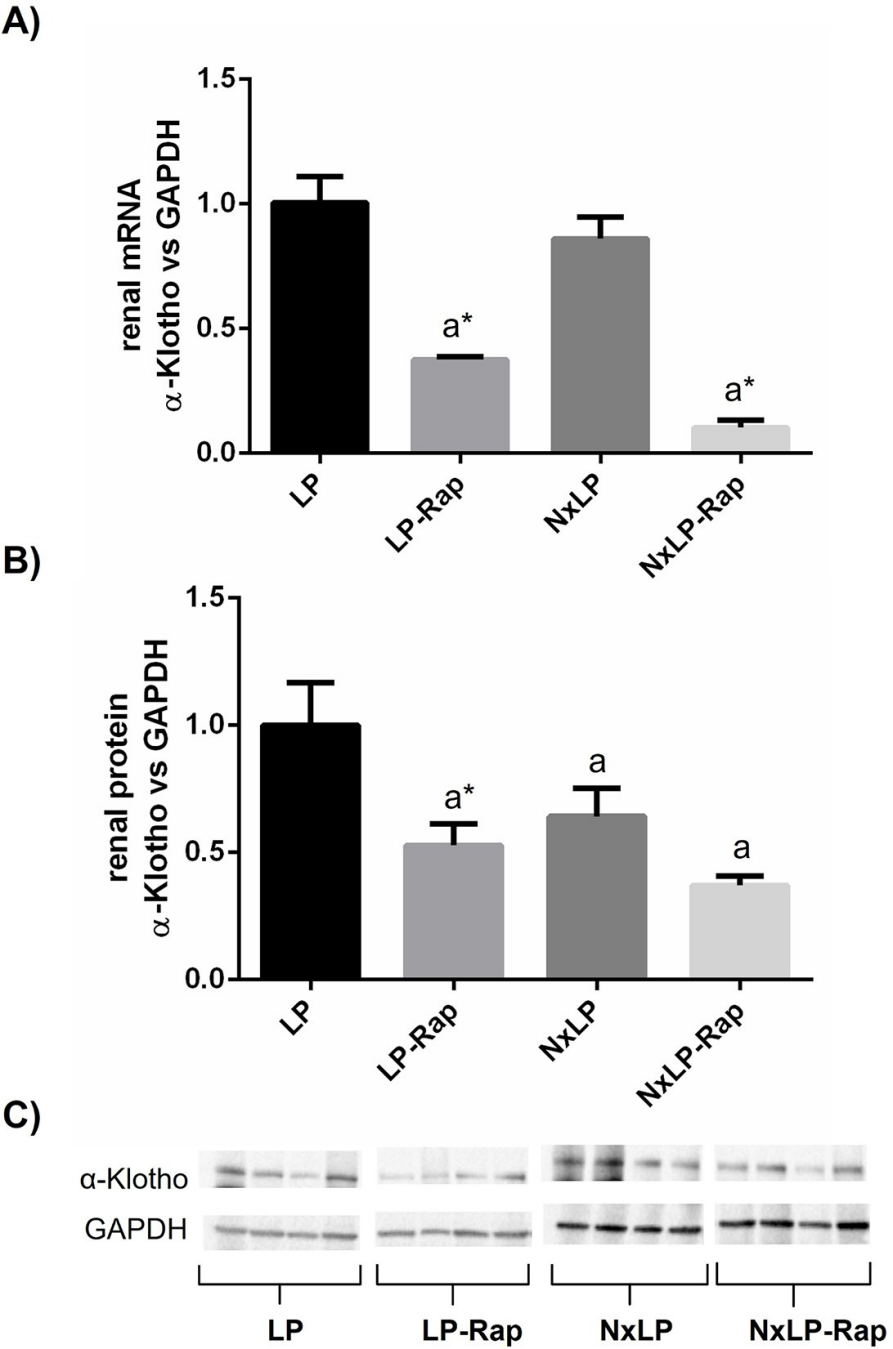
Renal expression of α-klotho at mRNA and protein level in rats fed low phosphorus. Renal expression of α-klotho at (A) mRNA and (B) protein level in rats fed 0.2% phosphorus with intact (NP) and reduced (NxNP) renal function receiving either placebo or rapamycin (Rap). ^a^*P*<0.05 vs NP; *P<0.05 vs its placebo counterpart. (C) Representative images of Western Blot (the complete Western Blot results are shown as Supplementary Data, S1 Raw images).

Rapamycin treatment reduced renal α-klotho expression in rats with intact and reduced renal function fed 0.2% P at both mRNA and protein levels. Thus, mRNA α-klotho/GADPH ratio was significantly lower, p = 0.0001, in LP-Rap rats, 0.4 ± 0.01 than in LP rats, 1.0 ± 0.1; and it was also lower, p<0.0001, in NxLP-Rap rats 0.1 ± 0.03 than in NxLP rats, 0.9 ± 0.1 (Fig 9A). At the protein level, rapamycin treatment only resulted in a significant reduction in α-klotho in rats with intact renal function, 0.5 ± 0.1 vs 1.0 ± 0.2, p = 0.01 (Fig 9B).

## Discussion

This study demonstrates downregulation of renal α-klotho expression in rats after treatment with rapamycin. The results were consistent both at the mRNA and protein levels and were observed in rats with intact and decreased renal function and with different dietary loads of P. The effect of rapamycin on klotho seems to be partly mediated by changes in phosphaturia.

Rapamycin is best known for its immunosuppressive effects [4] and for its influence on nutritional status and energy metabolism through inhibition of the mTORC1 complex [1, 2]. We observed a decrease in body weight gain in rats with normal renal function after treatment with rapamycin. Rapamycin has been shown to reduce body weight by inducing fat mass loss and thus decreasing adiposity in rats [25, 26]. Nephrectomized rats did not respond to rapamycin by reducing body weight gain, probably because they already had low body weight and very little fat mass (secondary to uremia). This has important clinical implications because, as previously reported by our laboratory [27], in the context of uremia a further decrease in body weight may be catastrophic in terms of survival.

Additionally, rapamycin also has an impact on mineral metabolism. In an elegant study, Kempe et al. demonstrated that rapamycin influences phosphate balance by promoting phosphaturia in healthy mice [13]. Although they administered rapamycin for only 3 days, their results are similar to what we have observed in rats with normal renal function. It is interesting to note that both studies have found a decrease in FGF23 without changes in PTH after treatment with rapamycin. The reduction in FGF23 concentrations may be a physiologic response to the decrease in tubular transport of P elicited by rapamycin [13]. In addition, low plasma FGF23 may also be influenced by a direct effect of rapamycin on bone [14]. We found an increase in urinary Ca excretion in rats treated with rapamycin. This was not reported by Kempe et al. [13] and the difference may be related to the more prolonged treatment in our study. The phosphaturic effect of rapamycin was also detected in rats with reduced renal function, as well as in rats fed diets with low P concentration. FGF23 decreased after rapamycin treatment in rats fed normal and low P but not in uremic rats, although this latter finding is difficult to interpret due to the very high values and the great variability of FGF23 measurements in Nx rats. Since P retention and elevated FGF23 concentrations are two major problems in renal patients [28, 29], the overall effect of rapamycin facilitating P excretion and decreasing FGF23 would be favourable in the context or kidney disease.

α-Klotho is a protein, mainly expressed in the kidneys, that is essential for the phosphaturic action of FGF23 and may have an important role in the pathogenesis of several kidney diseases [18, 19]. When designing this study our hypothesis was that treatment with rapamycin would increase renal α-klotho expression. This hypothesis was based on the fact that α-klotho has been reported to be upregulated in the vasculature of rats treated with rapamycin [30]. In addition, in a previous study we had found that caloric restriction, a condition that like rapamycin inhibits mTOR, resulted in an increase in renal α-klotho expression in rats [27].

The effect of rapamycin on renal α-klotho has been previously explored but the few reported data, which are somewhat contradictory, are difficult to interpret because in many instances rapamycin was administered together with other drugs. Han et al. investigated the effect of cyclosporine, tacrolimus, and rapamycin on renal α-klotho expression in mice. They found that both cyclosporine and tacrolimus decreased α-klotho but rapamycin did not. However, when rapamycin was added to either cyclosporine or tacrolimus a further decrease in α-klotho was identified [22]. Infante et al. reported that adding rapamycin to the immunosuppressive protocol of renal transplant patients resulted in an increase in soluble α-klotho [23]. In a similar line, Mizusaki et al. reported that rapamycin potentiated the increase in soluble α-klotho observed in renal patients after transplantation [24].

We observed a consistent decrease in renal α-klotho expression (mRNA and protein) after treatment with rapamycin, both in rats with intact and decreased renal function. A possible explanation for this unexpected finding may be related to the phosphaturic effect of rapamycin. An increased tubular load of P may lead to tubular injury through the formation of Ca-P microcrystals that bind to Toll-like receptor 4 (TLR4) [31]. In fact, increased phosphaturia has been reported to be an independent factor in the progression of renal disease [32]. Moreover, high tubular load of P has been shown to promote a decrease in renal α-klotho expression [32]. Thus, the increased urinary excretion of P after rapamycin treatment could be responsible for the decreased renal α-klotho. This contention is substantiated by the correlation study which shows a highly significant inverse correlation between urine P excretion and α-klotho expression.

To further investigate the role of urine P on the decrease in renal α-klotho the studies were repeated in rats fed low P diet. The results obtained in rats fed low P diet were practically identical to those previously recorded with normal P diet. Urinary excretion of P was very low in rats fed low P but was also increased by rapamycin. Again, a consistent decrease in renal α-klotho expression was detected in rats treated with rapamycin. Overall, these results would lend support to the hypothesis that the increase in urinary P down-regulates α-klotho. On the other hand, it seems clear that such regulation would not require a high level of P in urine, because even though urine P was increased when compared with placebo treated rats, it was still very low in the rats fed 0.2% P and treated with rapamycin.

It is interesting to note that renal α-klotho has been shown to increase in rats subjected to caloric restriction [27]. Caloric restriction, like rapamycin, is known to downregulate the mTOR pathway [16]. Both caloric restriction and rapamycin are associated with reduced body weight (actually, in our study rapamycin reduced body weight gain in rats with normal renal function). Since rapamycin is a very specific target of mTOR while caloric restriction influences many metabolic pathways, one of the conclusions of the present study must be that the previously reported effect of caloric restriction on renal α-klotho is likely to be independent of mTOR.

In conclusion rapamycin increases phosphaturia and down-regulates α-klotho expression in rats with normal and decreased renal function. These effects can be observed in animals ingesting normal and low P diet.

## Supporting information

S1 Fig Experimental design. Ca, calcium; LP, rats with intact renal function fed a low phosphorus diet; LP-Rap, rats with intact renal function fed a low phosphorus diet and treated with rapamycin; NP, rats with intact renal function fed a normal phosphorus diet; NP-Rap, rats with intact renal function fed a normal phosphorus diet and treated with rapamycin; NxLP, nephrectomized rats fed a low phosphorus diet; NxLP-Rap, nephrectomized rats fed a low phosphorus diet and treated with rapamycin, NxNP, nephrectomized rats fed a normal phosphorus diet; NxNP-Rap, nephrectomized rats fed a normal phosphorus diet and treated with rapamycin; P, phosphorus.
(TIF)

S2 Fig Correlation α-klotho vs phosphaturia. Scatterplot showing correlation between renal mRNA α-klotho expression and daily urinary excretion of phosphorus (P) in rats fed normal P diet.
(TIF)

S1 Raw images Original Western blots. Images of the unprocessed nitrocellulose membranes used for Western Blot analysis. LP, rats with intact renal function fed a low phosphorus diet; LP-Rap, rats with intact renal function fed a low phosphorus diet and treated with rapamycin; NP, rats with intact renal function fed a normal phosphorus diet; NP-Rap, rats with intact renal function fed a normal phosphorus diet and treated with rapamycin; NxLP, nephrectomized rats fed a low phosphorus diet; NxLP-Rap, nephrectomized rats fed a low phosphorus diet and treated with rapamycin, NxNP, nephrectomized rats fed a normal phosphorus diet; NxNP-Rap, nephrectomized rats fed a normal phosphorus diet and treated with rapamycin; P, phosphorus.
(PDF)

S1 Table Sequences of primers used for mRNA quantification by RT-PCR. (DOCX)
