# Supplementary figures and images for: Rapamycin downregulates α-klotho in the kidneys of female rats with normal and reduced renal function
Source: PLoS One. 2023 Nov 28;18(11):e0294791. doi: 10.1371/journal.pone.0294791 (PMC10684065; doi:10.1371/journal.pone.0294791)

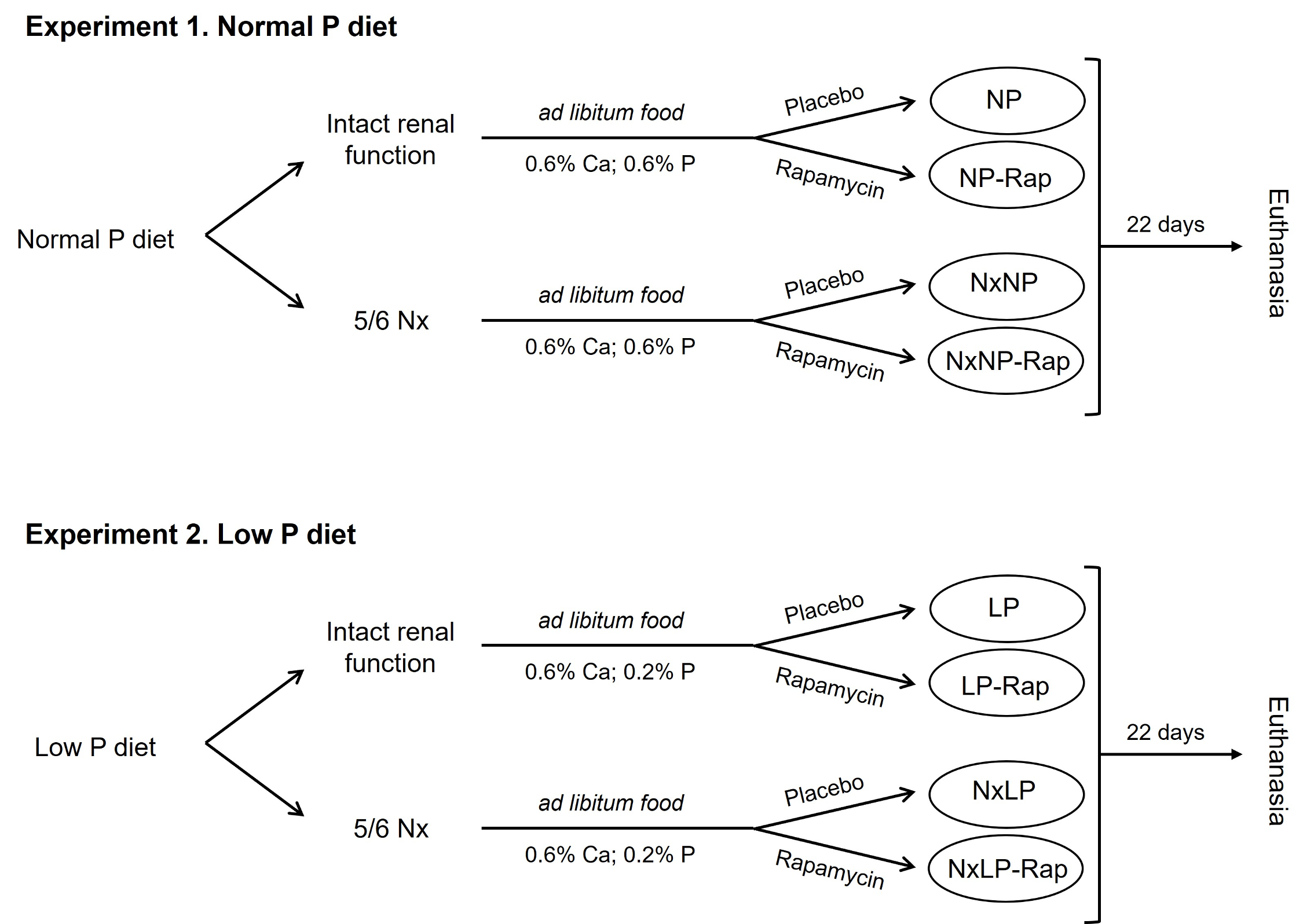

Supplement: S1 Fig — Ca, calcium; LP, rats with intact renal function fed a low phosphorus diet; LP-Rap, rats with intact renal function fed a low phosphorus diet and treated with rapamycin; NP, rats with intact renal function fed a normal phosphorus diet; NP-Rap, rats with intact renal function fed a normal phosphorus diet and treated with rapamycin; NxLP, nephrectomized rats fed a low phosphorus diet; NxLP-Rap, nephrectomized rats fed a low phosphorus diet and treated with rapamycin, NxNP, nephrectomized rats fed a normal phosphorus diet; NxNP-Rap, nephrectomized rats fed a normal phosphorus diet and treated with rapamycin; P, phosphorus. (TIF) [file pone.0294791.s001.tif]

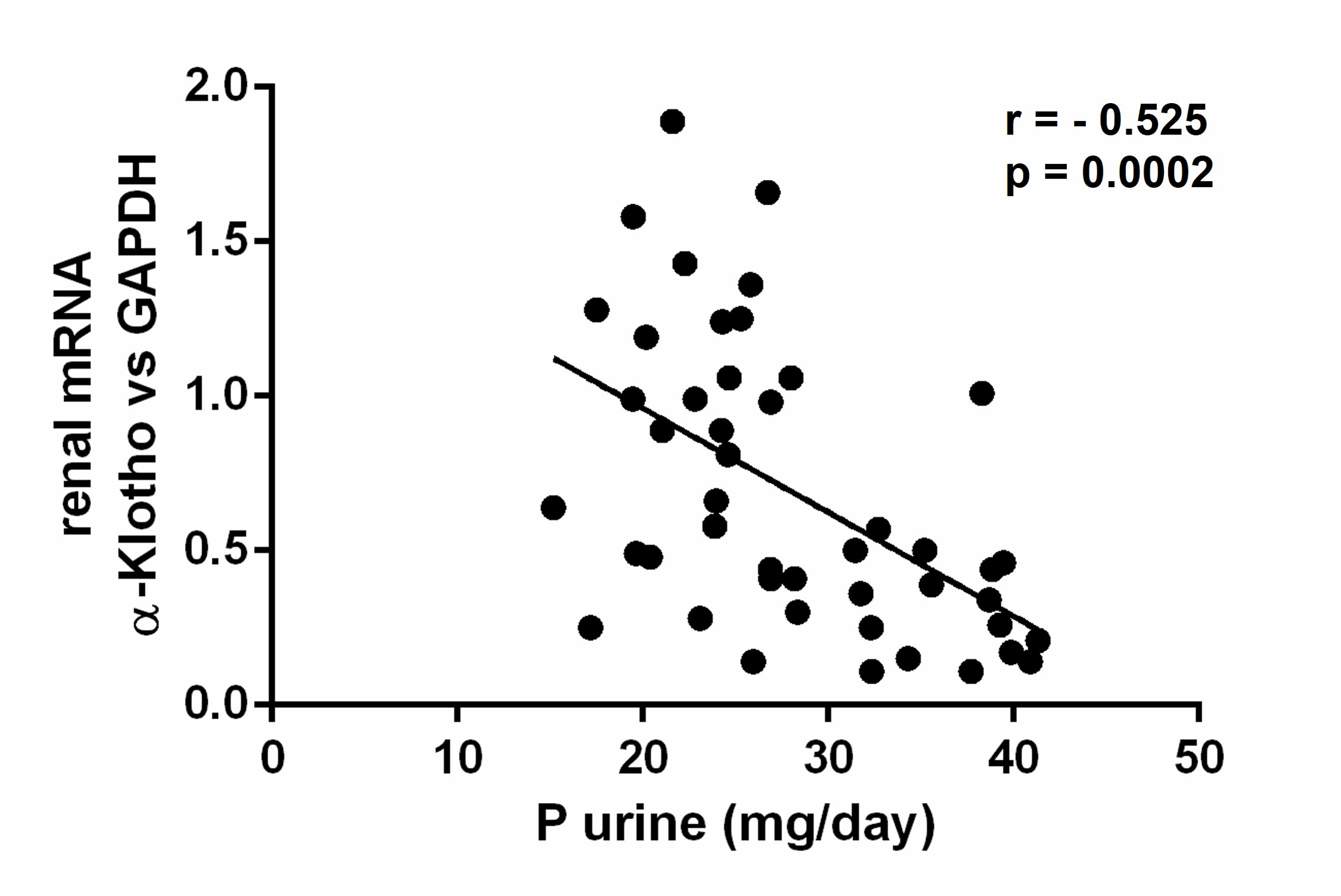

Supplement: S2 Fig — Scatterplot showing correlation between renal mRNA α-klotho expression and daily urinary excretion of phosphorus (P) in rats fed normal P diet. (TIF) [file pone.0294791.s002.tif]

### A) Experiments with Normal P diet

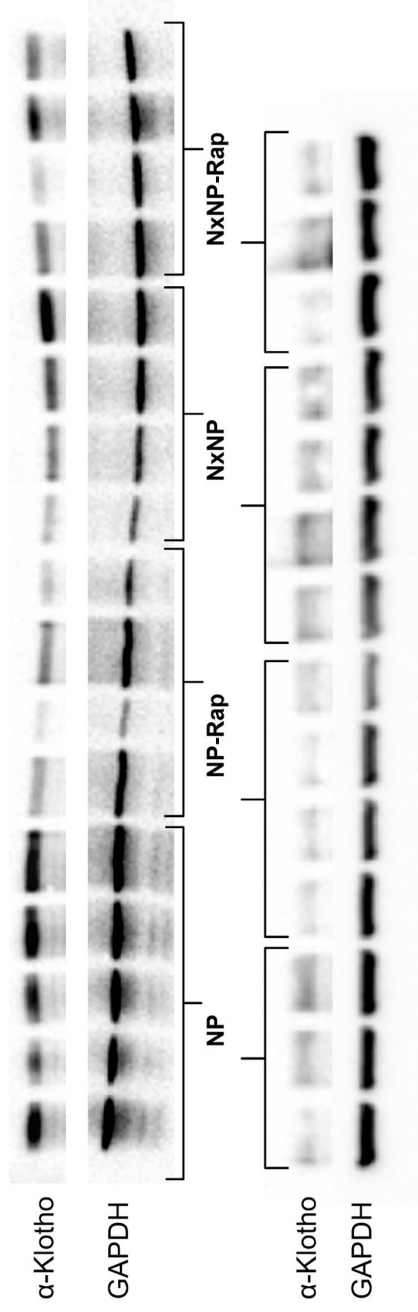

### B) Experiments with Low P diet

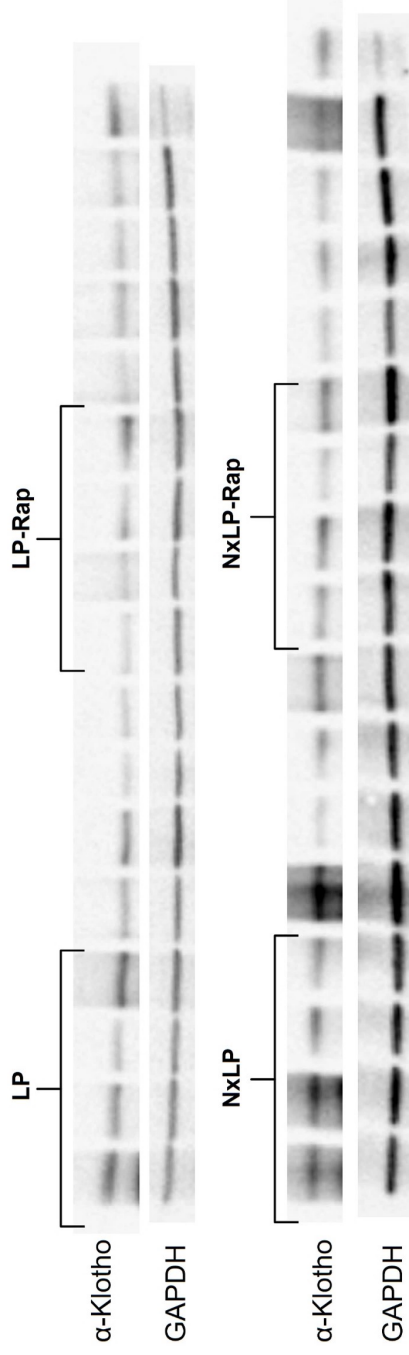

Supplement: S1 Raw images — Images of the unprocessed nitrocellulose membranes used for Western Blot analysis. LP, rats with intact renal function fed a low phosphorus diet; LP-Rap, rats with intact renal function fed a low phosphorus diet and treated with rapamycin; NP, rats with intact renal function fed a normal phosphorus diet; NP-Rap, rats with intact renal function fed a normal phosphorus diet and treated with rapamycin; NxLP, nephrectomized rats fed a low phosphorus diet; NxLP-Rap, nephrectomized rats fed a low phosphorus diet and treated with rapamycin, NxNP, nephrectomized rats fed a normal phosphorus diet; NxNP-Rap, nephrectomized rats fed a normal phosphorus diet and treated with rapamycin; P, phosphorus. (PDF) [file pone.0294791.s003.pdf]
