# Supplementary material for: Rapamycin downregulates α-klotho in the kidneys of female rats with normal and reduced renal function
Source: PLoS One. 2023 Nov 28;18(11):e0294791. doi: 10.1371/journal.pone.0294791 (PMC10684065; doi:10.1371/journal.pone.0294791)
Supplement: S1 Table — (DOCX) [file pone.0294791.s004.docx]

**S1 Table.** **Sequences of primers used for mRNA quantification by RT-PCR.**

| **Gene** | **Forward primer (5′-3′)** | **Reverse primer (5′-3′)** |
| --- | --- | --- |
| Rat GAPDH | AGGGCTGCCTTCTCTTGTGAC | TGGGTAGAATCATACTGGAACATGTAG |
| Rat α-klotho | CTCTGAAAGCCTACGTGTTGG | TAGAAACGAGATGAAGGCCAG |
| Human β-actin | GCA CTC TTCCAGCCTTCCTT | ATCCACATCTGCTGGAAGGT |
| Human α-klotho | CCACTCGAAACCATCCATGAG | GACCACCAAGAGAGATGATGC |

GAPDH, glyceraldehyde-3-phosphate dehydrogenase. All primers were purchased from Eurofins Genomics Germany GmbH, Ebersberg, Germany.
